# Results from the 5-year, phase IV RENEW (Registry to Evaluate Novantrone Effects in Worsening Multiple Sclerosis) study

**DOI:** 10.1186/1471-2377-13-80

**Published:** 2013-07-11

**Authors:** Victor M Rivera, Douglas R Jeffery, Bianca Weinstock-Guttman, Daena Bock, Fernando Dangond

**Affiliations:** 1 Baylor College of Medicine, Houston, TX, USA; 2 Cornerstone Health Care, Advance, NC, USA; 3 Jacobs Neurological Institute, State University of New York-University of Buffalo, Buffalo, NY, USA; 4 EMD Serono Inc, Rockland, MA, USA

## Abstract

**Background:**

Registry to Evaluate Novantrone Effects in Worsening Multiple Sclerosis (RENEW) was a 5-year, phase IV study in which the safety of Mitoxantrone was monitored in a patient cohort from the United States (US). The objective of the study was to evaluate the long-term safety profile of Mitoxantrone in patients with secondary progressive multiple sclerosis (SPMS), progressive relapsing multiple sclerosis (PRMS), and worsening relapsing-remitting multiple sclerosis (RRMS).

**Methods:**

Overall, 509 patients (395 SPMS, 81 worsening RRMS, 33 PRMS) were enrolled and treated at 46 multiple sclerosis (MS) treatment centers located in the US. Patients received Mitoxantrone in accordance with the package insert every 3 months. During the treatment phase, patients received laboratory workups and cardiac monitoring every 3 months and then annually for a total of 5 years.

**Results:**

Five hundred and nine subjects were enrolled in this trial and received at least one infusion of Mitoxantrone. Overall, 172 (33.8%) completed the 5-year trial (i.e., participated for 5 years ± 3 months [treatment + follow-up]); 337 (66.2%) did not complete the 5-year trial. Annual follow-up data were available for 250 of 509 enrolled patients. Left ventricular ejection fraction reduction under 50% was reported in 27 (5.3%) patients during the treatment phase (n = 509) and 14 (5.6%) patients during the annual follow-up phase (n = 250). Signs and symptoms of congestive heart failure were observed in 10 (2.0%) patients (six during treatment phase and four during the annual follow-up phase). Post-hoc analyses of the risk for cardiotoxicity outcomes revealed that cumulative dose exposure is the primary risk factor associated with the risk of cardiac toxicity with Mitoxantrone. Therapy-related leukemia was reported in three (0.6%) patients who received total cumulative Mitoxantrone doses of 73.5 mg/m^2^, 107.3 mg/m^2^, and 97.1 mg/m^2^ respectively. During the treatment phase, persistent amenorrhea developed in 22% (28/128) of women with regular menses and 51% (25/49) of women with irregular menses at baseline. During the annual follow-up phase, persistent amenorrhea developed in 5% (4/73) of women with regular menses at baseline.

**Conclusion:**

RENEW results are consistent with the known safety profile of Mitoxantrone, and provide additional long-term safety data for Mitoxantrone in MS patients.

## Background

Mitoxantrone is a synthetic anthracenedione agent originally developed for the treatment of cancer. Subsequent preclinical studies also demonstrated the immunosuppressive properties of Mitoxantrone [1-3], which eventually led to its clinical investigation in patients with multiple sclerosis (MS) [4,5]. Based on efficacy and safety results from two randomized clinical trials [4,5], Mitoxantrone was approved for the treatment of secondary progressive MS (SPMS), progressive relapsing MS (PRMS), and worsening relapsing-remitting MS (RRMS) in the United States (US) and Europe.

The use of Mitoxantrone in patients with MS, however, is associated with significant risks that include, among the more serious concerns, cardiotoxicity [6-15], treatment-related leukemia [16-32], and amenorrhea [33]. At present, the risk factors contributing to these toxicities are not completely understood. The main objective of the current report is to present the long-term safety data from the Registry to Evaluate Novantrone Effects in Worsening Multiple Sclerosis (RENEW) Study. Results from this phase IV study provide additional data on the long-term safety of Mitoxantrone in MS patients, and offer some insight into the risk factors underlying the development of Mitoxantrone-related toxicities.

## Methods

### Study design

RENEW was a multicenter, prospective, open-label, observational phase IV study monitoring the safety and tolerability of Mitoxantrone. From 17 October 2000 until 15 July 2008, 509 patients were enrolled and treated at 46 MS treatment centers located in the US.

Patients aged 18–65 years with clinically defined or a laboratory-supported diagnosis of SPMS, PRMS, or worsening RRMS were included in the study if they had a platelet count in excess of 100,000 cells/μL and a granulocyte count in excess of 2,000 cells/μL. Patients were excluded from the study if they presented with cardiac risk factors, including a history of congestive heart failure (CHF) or left ventricular ejection fraction (LVEF) <50% of normal.

Patients were treated with commercially available Mitoxantrone in accordance with the package insert (i.e., 12 mg/m^2^ as a 5–15 minute intravenous [IV] infusion). Doses were administered approximately every 3 months until a cumulative dose of 140 mg/m^2^ was reached, unless the patient or physician chose to discontinue or interrupt therapy or there was an adverse event (AE) that prevented further therapy. All enrolled patients, regardless of duration of therapy, were to be followed until the end of their 5-year trial participation period.

All participating investigators obtained local institutional review board approval prior to their initiation of the study, and all patients provided written informed consent before enrolment.

This study was conducted in compliance with the ethical principles enunciated in the World Medical Association's Declaration of Helsinki, Ethical Principles for Medical Research Involving Human Subjects.

### Study assessments

Patients were medically evaluated before treatment initiation, including the assessment of their Expanded Disability Status Scale (EDSS) score. Patients were assessed every 3 months during the treatment phase and then annually, for a total of 5 years. All enrolled subjects were to be followed to the end of their 5-year trial participation period.

Cardiovascular endpoints included LVEF (measured using echocardiogram or a multiple gated acquisition scan), symptoms of CHF, and cardiac-related serious AEs (SAEs). Assessments of LVEF and CHF were performed at baseline, before each infusion, and annually after treatment discontinuation or completion; SAEs were reported as they occurred.

At baseline, women were classified as having regular, irregular, or absent menses. Women with regular or irregular menses were monitored for the development of on-study persistent amenorrhea (defined as the absence of menses on at least two consecutive treatment visits that did not subsequently return during the course of the study) or transient amenorrhea (resumption of menses after absence on ≥2 consecutive treatment visits). Women with absent menses at baseline were monitored for resumption of menses (≥1 report of menses on-study).

### Statistical methods

Demographic, baseline, cumulative dose, and LVEF data were summarized by type of MS using descriptive statistics. Frequencies with 95% confidence intervals (CIs) were used to evaluate the incidence of SAEs, symptomatic CHF, abnormal LVEF (<50%), and serious infections. The incidence of clinical relapses, skipped and missed doses, abnormal laboratory values and concomitant medications were also summarized. For female patients, menstrual status was tabulated (regular, irregular, absent).

### Post-hoc analyses

To assess the factors potentially contributing to cardiotoxicity, post-hoc analyses were conducted in all patients receiving Mitoxantrone alone or concomitantly with other medications during the RENEW study. Patients with and without cardiac events were included in the analysis. Presence of cardiac events was defined as LVEF <50%, clinically significant drop in LVEF as indicated by a 10% decrease from baseline, cardiac SAE, or signs or symptoms of CHF requiring hospitalization or other treatment.

Poisson and logistic regression models were used to determine the significance of the effects of potential contributing factors on cardiotoxicity risk. A Fisher’s exact test was used to evaluate the effects of concomitant medication.

## Results

### Study cohort

Demographics, baseline characteristics, and study completion statistics for RENEW are shown in Table 1. Data were available for all 509 patients during the treatment phase. Long-term follow-up data were available for 250 (49%) patients (not all patients in the follow-up phase completed the study). In general, the majority of patients were female (67.6%); mean age at baseline was 46 years (range 19–68). Most patients (77.6%) were diagnosed with SPMS. The mean baseline LVEF was 62%. Median baseline EDSS score was 6 (range 0–9) and median times since onset of MS and MS diagnosis were 11.8 years (range 0.4–45.3) and 8.6 years (0.0–39.9), respectively.

**Table 1:** Patient characteristics

**Characteristic**	**Overall**	**Worsening**	**PRMS**	**SPMS**
		**RRMS**		
*At baseline*				
Patients enrolled,^a^ n (%)	509 (100.0)	81 (15.9)	33 (6.5)	395 (77.6)
Mean age, years (range)	46 (19–68)	40 (19–63)	47 (30–64)	47 (25–68)
Women,%	67.6	77.8	54.5	66.6
White,%	88.6	85.2	87.9	89.4
**History of MS, n**	**506**	**80**	**32**	**394**
Median EDSS score (range)	6.0 (0.0–9.0)	4 (1.0–8.0)	6.0 (1.5–8.5)	6.5 (0.0–9.0)
Median years since onset (range)	11.8 (0.4–45.3)	8.0 (0.4–29.2)	11.5 (0.6–34.5)	13.0 (0.6–45.3)
Median years since diagnosis (range)	8.6 (0.0–39.9)	4.8 (0.0–24.6)	7.3 (0.1–26.5)	9.3 (0.1–39.9)
Median years since most recent relapse (range)	0.4 (0.0–20.3)	0.2 (0.0–2.4)	0.2 (0.0–4.6)	0.5 (0.0–20.3)
Patients with no prior treatment for MS, n (%)	16 (3.1)	3 (3.7)	2 (6.1)	11 (2.8)
Women with regular menses, n (%)	128 (37.2)	34 (54.0)	7 (38.9)	87 (33.1)
**Cardiac, n**	**505**	**81**	**32**	**392**
Mean LVEF,% (range)	62 (50–83)	62 (50–83)	63 (52–79)	62 (50–83)
*Study completion, n*	*509*	*81*	*33*	*395*
Patients who did not complete	486 (95.5)	74 (91.4)	32 (97.0)	380 (96.2)
Mitoxantrone treatment,^b^ n (%)				
Did not complete 5-year study,^c^ n (%)	320 (62.9)	46 (56.8)	27 (81.8)	247 (62.5)
Completed 5-year study,^c^ n (%)	166 (32.6)	28 (34.6)	5 (15.2)	133 (33.7)
Patients who completed	23 (4.5)	7 (8.6)	1 (3.0)	15 (3.8)
Mitoxantrone treatment,^b^ n (%)				
Did not complete 5-year study,^c^ n (%)	17 (3.3)	5 (6.2)	1 (3.0)	11 (2.8)
Completed 5-year study,^c^ n (%)	6 (1.2)	2 (2.5)	0 (0)	4 (1.0)

EDSS=Expanded Disability Status Scale; LVEF=left ventricular ejection fraction;MS=multiple sclerosis; PRMS=progressive relapsing multiple sclerosis; RENEW=Registry to Evaluate Novantrone Effects in Worsening Multiple Sclerosis; RRMS=relapsing remitting multiple sclerosis; SPMS=secondary progressive multiple sclerosis.

^a^Patients with validated data who have received at least one dose of Mitoxantrone.

^b^Patients reaching a cumulative dose of 132 mg/m^2^ or greater are considered to have completed treatment.

^c^Patients participating in the study for 5 years ± 3 months are considered to have completed RENEW.

**Figure 1:**
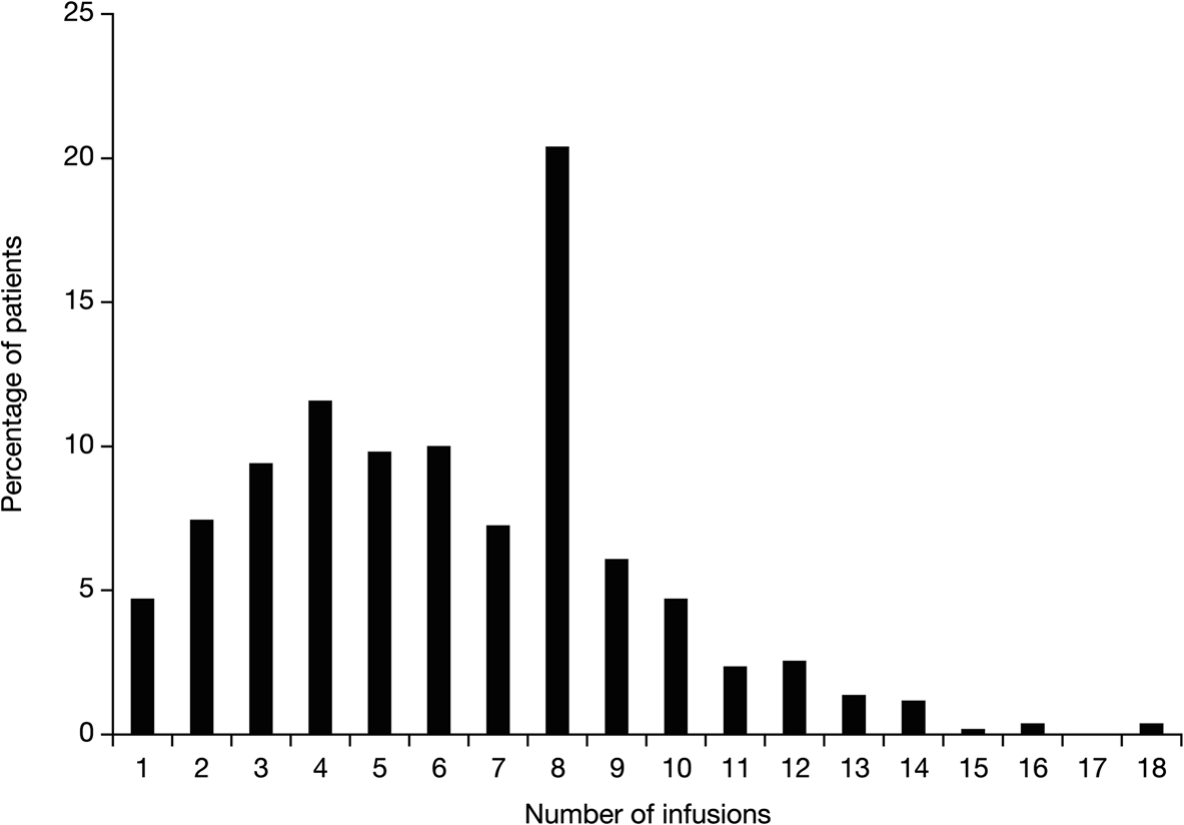
Distribution of the number of infusions of Mitoxantrone*. *Includes only patients who received at least one dose of Mitoxantrone.

Overall, 172 (33.8%) patients completed the RENEW study. Of these patients, six (1.2%) completed full treatment with Mitoxantrone (i.e., reached a cumulative dose of 132 mg/m^2^ or greater) while 166 (32.6%) patients did not. The mean duration of Mitoxantrone therapy was 1.5 years (range 0.0–4.9 years). The majority of subjects were in the trial for over 42 months (n = 263, 51.7%) while 57 (11.2%) patients were in the study for ≤12 months. The mean cumulative dose per patient was 69.8 mg/m^2^ (range 8.0–148.6 mg/m^2^). Patients received a mean of six infusions (range 1–18 infusions) during the study period (Figure 1). Doses <10 mg/m^2^ were received by 121 (24%) patients and accounted for 517 (16%) of all infusions administered during the study period. Overall, 361 out of 509 (71%) received concomitant therapy, of which the most common were glatiramer acetate (25.3%), methylprednisolone IV (21.0%), interferon beta-1a intramuscularly (20.6%), interferon beta-1b (14.9%), interferon beta-1a subcutaneous (11.8%), and oral prednisone (5.7%). Treatment discontinuation was reported in 486 (95.5%) patients (Table 2). The most frequently cited reasons for treatment discontinuation were physician decision (28.7%) and patient’s request (25.9%).

**Table 2:** RENEW treatment discontinuations

	**Overall**	**Type of MS**		
		**Worsening**	**PRMS**	**SPMS**
		**RRMS**		
Patients enrolled^a^	509	81	33	395
Discontinued treatment, n (%)	486 (95.5)	74 (91.4)	32 (97.0)	380 (96.2)
Death^b^	4 (0.8)	1 (1.2)	0	3 (0.8)
Other adverse event	14 (2.8)	2 (2.5)	2 (6.1)	10 (2.5)
Physician decision	146 (28.7)	28 (34.6)	6 (18.2)	112 (28.4)
Reached maximum cumulative dose (≥140 mg/m^2^)^c^	23 (4.5)	5 (6.2)	1 (3.0)	17 (4.3)
Lack of efficacy	16 (3.1)	0	2 (6.1)	14 (3.5)
Lost to follow-up	40 (7.9)	3 (3.7)	2 (6.1)	35 (8.9)
LVEF <50%	25 (4.9)	0	1 (3.0)	24 (6.1)
Clinically significant decrease in LVEF or occurrence of CHF^d^	10 (2.0)	2 (2.5)	2 (6.1)	6 (1.5)
Patient request	132 (25.9)	24 (29.6)	11 (33.3)	97 (24.6)
Other	98 (19.3)	16 (19.8)	6 (18.2)	76 (19.2)
Unknown	1 (0.2)	0	0	1 (0.3)

CHF=congestive heart failure; LVEF=left ventricular ejection fraction; MS=multiple sclerosis; PRMS=progressive relapsing multiple sclerosis; RENEW=Registry to Evaluate Novantrone Effects in Worsening Multiple Sclerosis; RRMS=relapsing-remitting multiple sclerosis; SPMS=secondary progressive multiple sclerosis.

^a^Patients with validated data who have received at least one dose of Mitoxantrone.

^b^Eight additional patients died following discontinuation; the reasons for discontinuation of Mitoxantrone treatment were: physician discretion (n = 4); patient request (n = 2); LVEF <50% (n = 1); and clinically significant reduction in LVEF/CHF (n = 1).

^c^Includes patients who have reached a cumulative dose of at least 132 mg/m^2^.

^d^Clinical significance determined by the study site investigator.

### Cardiovascular AEs

**Figure 2:**
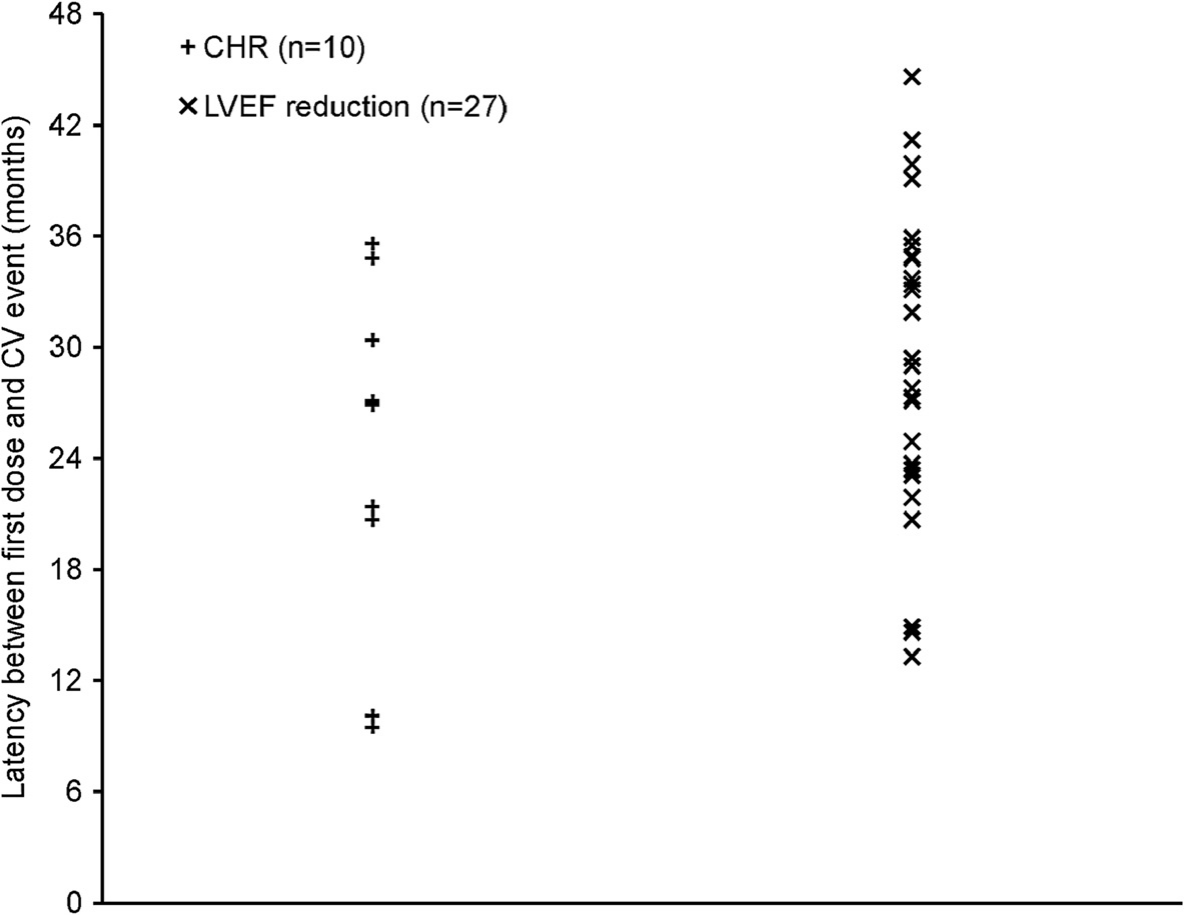
**Latency of CHF events and LVEF <50% events.** CHF=congestive heart failure; CV=cardiovascular; LVEF=left ventricular ejection fraction.

Ten patients experienced CHF during the trial (six during the treatment phase and four during the annual follow-up phase). The median latency of CHF events from the first dose of Mitoxantrone was 24.2 months (range 9.5–35.6 months) (Figure 2). Twenty-seven of 509 patients (5.3%) during the treatment phase and 14 of 250 patients (5.6%) during the annual follow-up phase experienced LVEF test results that decreased to below 50%. The median latency of LVEF reductions below 50% from the first dose of Mitoxantrone during the treatment phase was 29 months (range 13.3–44.6 months) (Figure 2). Twenty-five cardiac-related SAEs were reported among 22 of 509 patients (4.3%) during the treatment phase, with the most common being ejection fraction decreased (12 patients, 2.4%; 12 events) (Table 3). During the annual follow-up phase, five cardiac-related SAEs were reported in four of 250 patients (1.6%). These events included ejection fraction decreased (two patients, 0.8%; two events), cardiomyopathy (two patients; 0.8%; two events), and tachycardia (one patient, 0.4%; one event) (Table 3).

**Table 3:** Summary of cardiac-related SAEs

**SAE, n (%)**	**Overall**	**Treatment phase**	**Annual follow-up**
	**(N = 509)**	**(n = 509)**	**phase (n = 250)**
Any cardiac event	25^a^ (4.9)	22 (4.3)	4 (1.6)
Reduced LVEF	14 (2.8)	12 (2.4)	2 (0.8)
Myocardial infarction	4 (0.8)	4 (0.8)	0
Cardiomyopathy	5 (1.0)	3 (0.6)	2 (0.8)
Congestive heart failure	2 (0.4)	2 (0.4)	0
Cardiorespiratory arrest	1 (0.2)	1 (0.2)	0
Mitral valve incompetence	1 (0.2)	1 (0.2)	0
Tachycardia	2 (0.4)	1 (0.2)	1 (0.4)
Ventricular hypokinesia	1 (0.2)	1 (0.2)	0

LVEF=left ventricular ejection fraction; SAE=serious adverse event.

^a^One patient had an SAE in both the treatment and follow-up phase.

**Figure 3:**
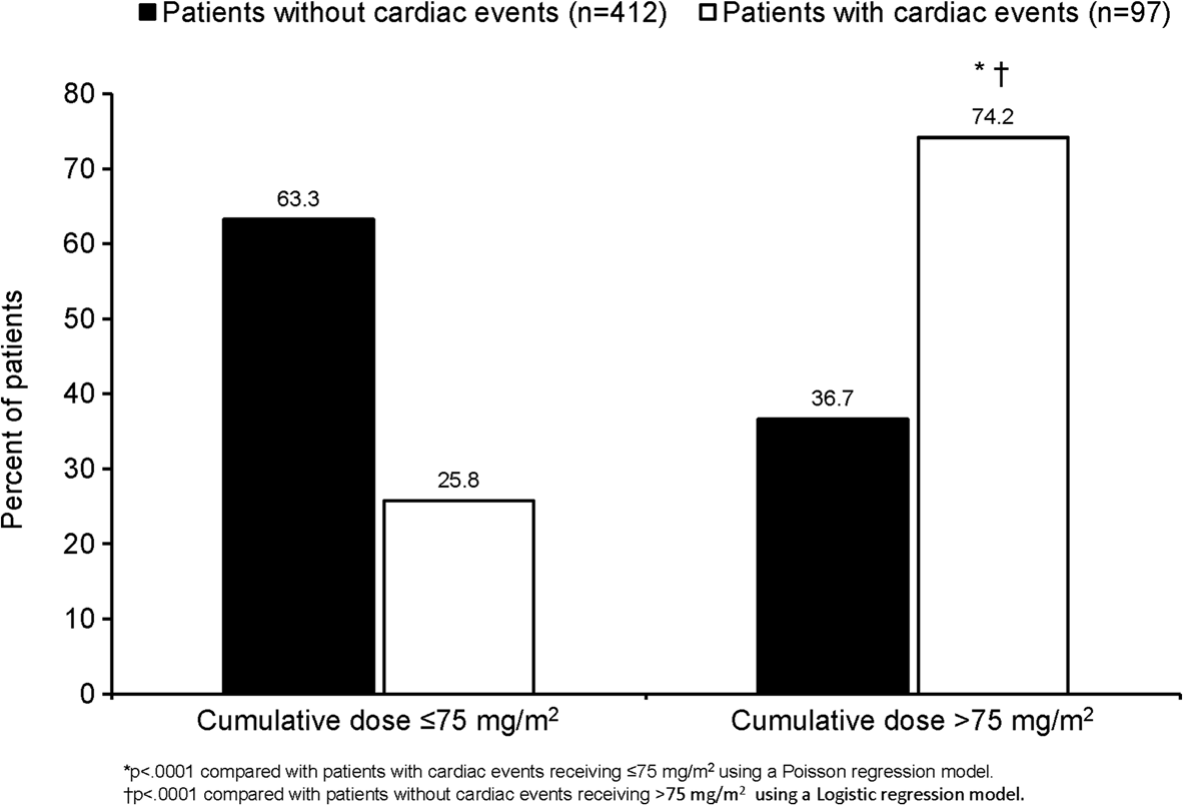
**Association between cardiotoxicity risk and cumulative dose.** Cardiac events includes patients with reported cardiac SAEs, with signs or symptoms of CHF requiring hospitalization or other therapy, with an LVEF decrease to 50% of baseline, or with a clinically significant drop in LVEF as indicated by a 10% decrease from baseline. CHF=congestive heart failure; LVEF=left ventricular ejection fraction; SAE=serious adverse event.

**Figure 4:**
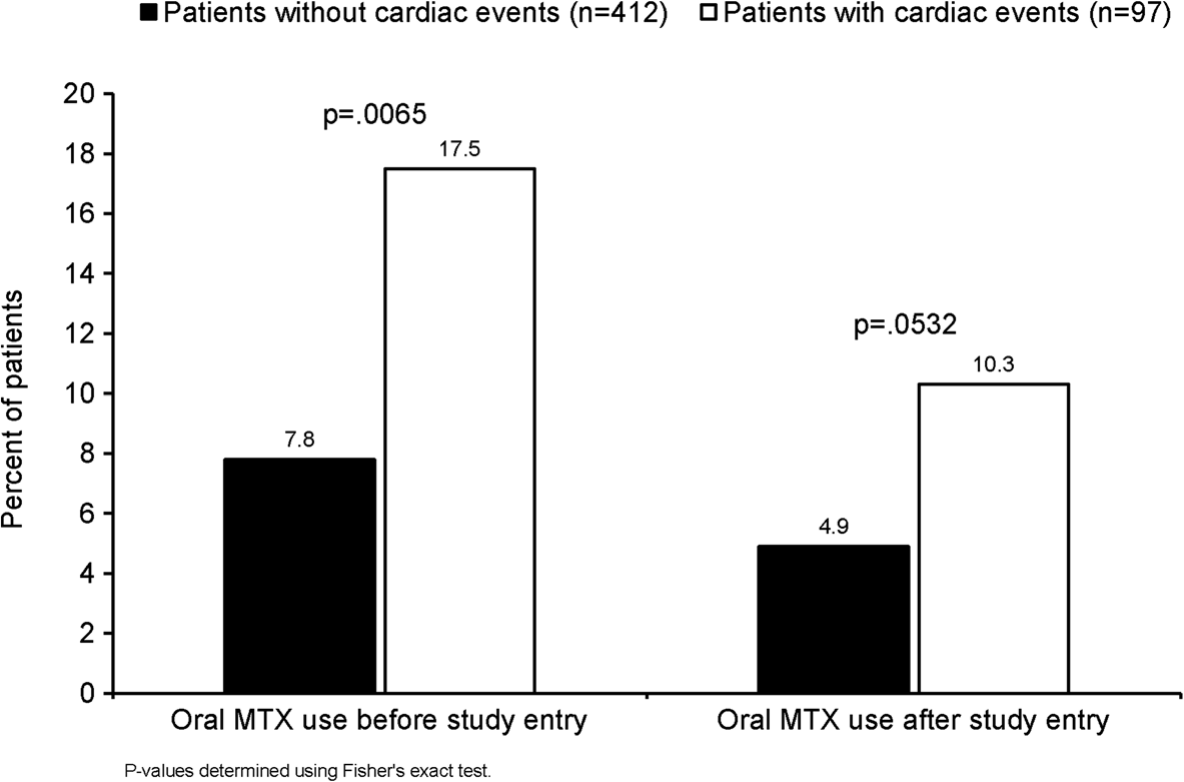
**Risk of cardiotoxicity with prior and/or concomitant Methotrexate therapy.** Cardiac events includes patients with reported cardiac SAEs, with signs or symptoms of CHF requiring hospitalization or other therapy, with an LVEF decrease to 50% of baseline, or with a clinically significant drop in LVEF as indicated by a 10% decrease from baseline. CHF=congestive heart failure; LVEF=left ventricular ejection fraction; MTX=Methotrexate; SAE=serious adverse event.

In response to a Food and Drug Administration request, additional post-hoc analyses of the RENEW study were completed. These analyses were intended to provide valid estimates of the risk for cardiotoxicity outcomes in the RENEW study, and to examine the impact of potential explanatory factors on cardiotoxicity risk among this cohort of MS patients. Results showed that the cumulative dose of Mitoxantrone is the primary risk factor associated with cardiotoxicity (Table 4). The mean cumulative dose was higher in patients with cardiac events (n = 97; 93.9 mg/m^2^) than in those without cardiac events (n = 412; 63.7 mg/m^2^). The dose-dependent, cardiotoxic effect of Mitoxantrone was confirmed to be statistically significant when cumulative dose was analyzed as a dichotomous covariate (Figure 3). Among patients with cardiac events, 74.2% of patients received doses >75 mg/m^2^ compared with 25.8% of patients who received ≤75 mg/m^2^ (p < 0.0001). Though not statistically significant, there were consistent trends suggesting higher cardiac toxicity in various populations, including: higher age group; SPMS vs relapsing MS; longer disease duration; higher EDSS; use of concomitant immunosuppressants and/or disease-modifying drugs (see Additional file 1). Lastly, use of Mitoxantrone and previous or concomitant use of oral Methotrexate was significantly associated with cardiotoxicity risk and there was a trend toward significance when oral Methotrexate was used after study entry (Figure 4).

**Table 4:** Association between cardiotoxicity risk and cumulative dose

**Cumulative dose**	**Overall**	**Patients with**	**Patients without**	**p value**^**a**^
**category, n (%)**	**(N = 509)**	**cardiac events**	**cardiac events**	
		**(n = 97)**	**(n = 412)**	
>0 and ≤35 mg/m^2^	82 (16.1)	6 (6.2)	76 (18.4)	REF
>35 and ≤75 mg/m^2^	204 (40.1)	19 (19.6)	185 (44.9)	>0.05
>75 and ≤105 mg/m^2^	149 (29.3)	33 (34.0)	116 (28.2)	<0.01
>105 mg/m^2^	74 (14.5)	39 (40.2)	35 (8.5)	<0.0001

^a^From Poisson and logistic regression models; pairwise comparisons of cumulative dose categories are presented with lowest dose category as reference.

REF=reference value.

### Hematologic effects

Leukemia was reported in three patients, resulting in an incidence rate of 0.6%. Among these cases, two occurred during the annual follow-up phase of the study. In case 1, the patient was diagnosed with acute myelogenous leukemia (AML), M4 type in 2004, approximately 30 months following the first dose of Mitoxantrone (total cumulative dose was 73.5 mg/m^2^). The patient received chemotherapy and achieved remission in November 2005. As of January 2008, the patient was stable with no recurrence. In case 2, the patient was diagnosed with chronic phase chronic myeloid leukemia (CML) in 2006, approximately 63 months following the first dose of Mitoxantrone (total cumulative dose was 107.3 mg/m^2^). In June 2006 (exact date unknown), the patient began therapy with imatinib. On 28 June 2006, the patient’s white blood cell count fell from 30,000 to 18,900 and the event was not resolved. No additional information regarding the outcome of case 2 is available at this time. Case 3 involved a patient who developed acute promyelocytic leukemia (APL). The total cumulative dose of Mitoxantrone was 97.1 mg/m^2^ (first dose on 29 December 2000 and last dose on 3 October 2003). Details regarding the date of APL diagnosis and outcome were not included in the initial report. Three requests for information were made to the trial site to obtain details surrounding the diagnosis of APL (including pathology reports) and subsequent treatment and outcome, but no further information on the patient’s leukemia was obtainable.

### Amenorrhea

At baseline, 128 women reported regular menses. During the treatment phase of the study, 28 (22%) women developed persistent amenorrhea having received a mean cumulative dose of 52.1 mg/m^2^, and five (4%) women developed transient amenorrhea having received a mean cumulative dose of 70.5 mg/m^2^. During the annual follow-up phase, 73 women reported regular menses at baseline; of these women, four (5%) developed persistent amenorrhea while one (1%) developed transient amenorrhea.

Forty-nine women reported irregular menses at baseline; of these women, 25 (51%) developed persistent amenorrhea having received a mean cumulative dose of 38.2 mg/m^2^during the treatment phase of the study, and one (2%) developed transient amenorrhea having received a cumulative dose of 59 mg/m^2^. During the annual follow-up phase, no patients with irregular menses at baseline developed persistent amenorrhea while only one of 26 women (4%) developed transient amenorrhea. During the treatment phase of the study, resumption of menses was reported by three women (1.8%) who received a cumulative dose of 23 mg/m^2^ and had reported an absence of menses at baseline; no patients with absent menses at baseline resumed menses during the observational phase.

### AEs and deaths

During the treatment phase, a total of 143 SAEs were reported in 88 patients (see Table 5 and Additional file 2). The most commonly reported SAE during the treatment phase was decreased ejection fraction (12 patients, 2.4%; 12 events, 11 of which were considered treatment related). During the annual follow-up phase of the study, 23 patients experienced 39 SAEs (see Table 6 and Additional file 2). The most commonly reported SAE during the annual follow-up phase was urinary tract infection (UTI) (three patients, 1.2%; four events).

**Table 5:** Treatment-related SAEs during RENEW treatment phase

**SAE**	**Treatment phase (n = 509)**		
	**Number of treatment-related events**		
	**Possible**	**Probable**	**Total**
Decreased ejection fraction	1	10	11
Urinary tract infection	1	3	4
Cardiomyopathy	0	3	3
Febrile neutropenia	1	2	3
Leukopenia	0	3	3
Pneumonia	0	3	3
Congestive heart failure	0	2	2
Cellulitis gangrenous	0	1	1
Herpes zoster	0	1	1
Upper respiratory tract infection	0	1	1
Urosepsis	0	1	1
Ventricular hypokinesia	0	1	1
Myocardial infarction	2	0	2
Nausea	2	0	2
Sepsis	2	0	2
Septic shock	2	0	2
Vomiting	2	0	2
Abortion incomplete	1	0	1
Cellulitis	1	0	1
Deep vein thrombosis	1	0	1
Dizziness	1	0	1
Fungal skin infection	1	0	1
Hydronephrosis	1	0	1
Localized infection	1	0	1
Lung infection	1	0	1
Meningitis	1	0	1
Pituitary tumor	1	0	1
Pulmonary embolism	1	0	1
Varicella	1	0	1

SAE=serious adverse event; RENEW=Registry to Evaluate Novantrone Effects in Worsening Multiple Sclerosis.

**Table 6:** Treatment-related SAEs during RENEW annual follow-up phase

**SAE**	**Annual follow-up phase (n = 250)**		
	**Number of treatment-related events**		
	**Possible**	**Probable**	**Total**
Decreased ejection fraction	0	2	2
Acute myeloid leukemia	0	1	1
Pneumonia	0	1	1
Anorexia	1	0	1
Cardiomyopathy	1	0	1
Tachycardia	1	0	1
Chronic myeloid leukemia	1	0	1
Endometrial cancer	1	0	1
Carotid artery occlusion	1	0	1
Cerebrovascular accident	1	0	1
Dyspnea	1	0	1

SAE=serious adverse event; RENEW=Registry to Evaluate Novantrone Effects in Worsening Multiple Sclerosis.

A total of 60 cases of serious infection were reported (n = 41) during the study (see Additional file 3). During the treatment phase, 38 (7.5%) patients had 47 infectious SAEs, with the most common being UTI (11 patients, 2.2%; 13 events). During the annual follow-up phase, eight (3.2%) patients had 13 serious infectious events, with the most common being UTI (three patients, 1.2%; four events).

Twelve deaths were reported during the study (Table 7). Eight of the 12 deaths occurred during the treatment phase. Median EDSS score was 7 (range 4–9); median cumulative dose was 81.9 mg/m^2^ (range 11.1–119.4 mg/m^2^). Events accounting for at least two deaths included: infection (n = 3; pneumonia, acute meningitis, and septic shock); cardiac-related SAEs (n = 2), and pulmonary embolism (n = 2).

**Table 7:** Deaths during the RENEW study

**Count**	**Age (years)/**	**Form**	**Baseline**	**Cause(s)**	**Study phase**	**Treatment**	**Cumulative**
	**gender**	**of MS**	**EDSS**			**related?**	**dose mg/m**^**2**^
1	51/F	SPMS	7	Pneumonia	Treatment	No	91.3
2	43/F	SPMS	6	Decreased ejection fraction,	Treatment	Probable	96.8
				cardiomyopathy, congestive heart			
				failure			
3	50/F	SPMS	8.5	Pulmonary embolism	Treatment	No	12.2
4	57/M	SPMS	7	Road traffic accident	Annual follow-up	No	99.7
5	57/M	SPMS	6	Prostate cancer	Treatment	No	Unknown
6	49/M	SPMS	6.5	Cerebrovascular accident, carotid	Annual follow-up	Possible	119.4
				artery occlusion			
7	57/M	PRMS	7	Pulmonary edema	Annual follow-up	Unknown	109.5
8	55/F	SPMS	6.5	Pulmonary embolism	Annual follow-up	No	67.7
9	51/F	RRMS	4	Acute meningitis	Treatment	Possible	11.9
10	68/M	SPMS	7.5	Respiratory failure	Treatment	No	11.1
11	37/F	SPMS	9	Cardiopulmonary arrest	Treatment	No	18.4
12	44/F	SPMS	7	Septic shock	Treatment	Possible	81.9

EDSS=Expanded Disability Status Scale; F=female; M=male; MS=multiple sclerosis; PRMS=progressive relapsing multiple sclerosis; RENEW=Registry to Evaluate Novantrone Effects in Worsening Multiple Sclerosis; SPMS=secondary progressive multiple sclerosis.

## Discussion

The cardiotoxic risk associated with Mitoxantrone in patients with MS is well-documented [6-15]. In 2010, the Therapeutics and Technology Assessment (TTA) Subcommittee of the American Academy of Neurology reviewed all articles and abstracts published before July 2009 containing ‘Mitoxantrone’ and ‘multiple sclerosis’ [25]. Investigators identified and consolidated 11 class III evidence studies to analyze cardiotoxicity. From this analysis (n = 716), decreased LVEF was estimated to be approximately 12% while the risk of CHF was estimated to be approximately 0.4%; the number needed to harm (NNH) for left ventricular dysfunction was eight. The authors cautioned, however, that these estimates are a general approximation given that dosing regimens and cardiac testing varied among the different treatment centers. More recently, Le Page and colleagues published final results from a 5-year prospective study in a French cohort of 802 MS patients [33]. The final mean cumulative dose of Mitoxantrone in this study was 78 mg/m^2^, with 18.5% of patients receiving more than 100 mg/m^2^; the mean follow-up was 6.7 years. There was one case of acute CHF out of the 802 total patients (0.1%). Of 794 patients who received LVEF assessment testing, 39 (4.9%) experienced an asymptomatic decrease of LVEF <50%. Decreased LVEF <50% was reversible in 27 (69.2%) patients, persistent in 11 (28.2%) patients, and only observed on the last scan in one patient (2.6%) remaining clinically symptomatic. Analysis of risk factors did not reveal any factor predictive of the occurrence of a LVEF decrease.

In the RENEW trial, the mean cumulative dose of Mitoxantrone was 69.8 mg/m^2^ and patients received treatment for an average of 1.5 years. During the treatment (N = 509) and follow-up (n = 250) phases, LVEF <50% occurred in 5.3% and 5.6%, respectively; CHF was observed in 10 (2%) patients overall. The decreased LVEF rates reported here are comparable to those reported in the French cohort and below the estimate reported by the TTA Subcommittee. Conversely, the rate of CHF in RENEW appears to be significantly higher (at least 5-fold) compared with either TTA Subcommittee estimates or the French cohort. It should be noted, however, that confirmation of CHF was not required in patients who experienced signs or symptoms of CHF in the RENEW study.

Post-hoc analyses of risk factors revealed that the cumulative dose of Mitoxantrone is the primary risk factor associated with cardiotoxicity in RENEW. There were also consistent trends suggesting higher cardiac toxicity in various sub-populations of patients, including: higher age group, SPMS vs relapsing MS, longer disease duration, higher EDSS, use of concomitant immunosuppressants and/or disease-modifying drugs. The independent contribution of any one of these individual demographic and disease characteristics to the risk of cardiotoxicity needs to be interpreted with caution since they may be interrelated and therefore coexist in the same patient (e.g., an older subject will have had MS for a longer period of time and may have accumulated a higher EDSS score, leading to attempts by clinicians to add other drugs (i.e., immunosuppressants and/or disease-modifying drugs)). The independent contribution of any of these factors to a higher predisposition to risk of cardiac toxicity with Mitoxantrone is unknown. Results also suggested that previous or concomitant treatment with oral Methotrexate may also increase the risk of cardiotoxicity. The cardiovascular risks associated with Methotrexate, however, have yet to be established in patients with MS. The majority of studies evaluating the impact of Methotrexate on cardiovascular events have focused on patients with rheumatoid arthritis (RA), a systemic inflammatory disease associated with an increased risk for morbidity and mortality from cardiovascular disease [34]. Analysis in prospective [35], observational [36], and retrospective [37] studies have suggested that Methotrexate is cardioprotective in RA patients. Conversely, a longitudinal cohort study analyzing data from 10,156 patients enrolled in the Consortium of Rheumatology Researchers of North America Rheumatoid Arthritis registry reported that Methotrexate was not associated with a reduced risk of the primary composite cardiovascular endpoint of incident non-fatal myocardial infarction, non-fatal stroke or transient ischemic attack, and cardiovascular-related death [38]. Controversy exists on whether chronic MS is associated with cardiovascular disease, with studies suggesting an association [39] and others rejecting it [40,41]. The association between Methotrexate and cardiotoxicity observed in RENEW remains speculative since analysis was post-hoc rather than pre-specified. Furthermore, analysis with a larger cohort of patients is also required to confirm the role of age and Methotrexate therapy as predictive factors of cardiotoxicity.

Reports from Spanish cohorts have raised concerns about the increased risk of leukemia in MS patients treated with Mitoxantrone [17,28]. In the report by Pascual and colleagues [28], investigators reported results from two separate Spanish cohorts. In the first cohort treated with Mitoxantrone [17,28], there were four cases of leukemia among 142 patients with RRMS and SPMS. The four patients received total Mitoxantrone doses of 30, 60, 70, and 100 mg/m^2^. The cumulative incidence of leukemia was 2.82% (95% CI, 1.2–4.4) over a median follow-up time of 40 months since the start of treatment. In the second cohort containing 88 patients with MS [28], there were two cases of leukemia in patients receiving 137 mg/m^2^ and 159 mg/m^2^ of Mitoxantrone, respectively. The cumulative incidence of treatment-related leukemia in this cohort was 2.27% (95% CI, 1.1–3.4).

In contrast, analysis of leukemia among the French cohort identified two cases of Mitoxantrone-related leukemia to yield an incidence of 0.25%. The total cumulative dose of Mitoxantrone was 70 mg/m^2^ in each case [33]. In addition, retrospective analysis of data from 40 Italian MS centers identified 30 cases of AML among 3,220 patients treated with Mitoxantrone, yielding an incidence of 0.93%. Furthermore, the authors reported that MS patients presenting with AML received a significantly higher dose of Mitoxantrone compared with their non-AML counterparts (78.3 vs 64.7 mg/m^2^, respectively; p = 0.028) [26].

In the RENEW study, there were three reports of Mitoxantrone-related leukemia (AML and CML) among the 509 patients (incidence of 0.6%). The total cumulative dose was 73.5 mg/m^2^, 107.3 mg/m^2^, and 97.1 mg/m^2^for each case. These results are consistent with results from the French and Italian cohort studies. RENEW results are also consistent with a TTA Subcommittee analysis of treatment-related leukemia among 4,076 patients combined from 32 class III or IV evidence studies [25]. According to TTA estimates, the incidence of treatment-related leukemia in patients with MS is approximately 0.81%, with a NNH of 123. Again, the authors caution that this is a general approximation given the variability in time of follow-up among the different studies, especially case reports.

The disparity in incidence rates between RENEW/French cohort and the Spanish cohort is not completely understood. It is worth noting, however, that the mean cumulative dose was 87.7 mg/m^2^ when the 10 patients from the respective cohorts are combined. Given the dose-dependent data reported by the Italian group [26], it is reasonable to speculate that the 10 patients from the combined cohorts were at higher risk for developing treatment-related leukemia by virtue of their cumulative dose. Genetic risk cannot be ruled out either.

The French cohort evaluated the reproductive safety profile of Mitoxantrone in 317 women with MS starting treatment at 45 years of age or younger [33]. Transient amenorrhea was observed in 27% of women while persistent amenorrhea was observed in 17.3% of women. No persistent amenorrhea, however, was observed in women treated before the age of 25. The likelihood of experiencing persistent amenorrhea increased with age in this cohort. In the RENEW trial, the incidence of transient amenorrhea (4% during the treatment phase and 1% during follow-up) was lower compared with the French cohort. The incidence of persistent amenorrhea was consistent between studies (22% during the treatment phase and 5% during follow-up in RENEW). In the RENEW cohort, women who at baseline reported irregular menses had a higher incidence (51%) of persistent amenorrhea compared to women who reported regular menses (22%), despite the fact that the mean cumulative dose of Mitoxantrone was higher in the latter group. These results indicate that women with menstrual irregularity are at higher risk of developing persistent amenorrhea while taking Mitoxantrone.

## Conclusion

RENEW provides additional data on the long-term safety of Mitoxantrone in patients with worsening MS. There are limitations that must be considered when evaluating this data. First, RENEW data are registry-based, and not from a controlled trial. As a result, more than half of the patients were lost during the 5-year planned follow-up. Patient drop out, therefore, may lead to underestimation of the incidence of cardiovascular events, leukemia, and SAEs reported here. To ascertain whether there are more cases of cardiovascular events or leukemia, a second RENEW Study has been initiated to contact patients who originally left RENEW for reasons other than death or lost to follow-up. Another factor that may lead to underestimation of Mitoxantrone-related SAEs includes the protocol for evaluating SAE by relationship to study drug. In RENEW, SAE by relationship to study drug was determined by the local study investigator rather than an independent review board. Lastly, there is also complexity in data interpretation due to variability in physician behaviour/use of Mitoxantrone (on-label vs off-label) and variability in severity of patient disease when first treated with Mitoxantrone.

Taking study limitations into consideration, the data reported here demonstrate that, consistent with previous reports, Mitoxantrone therapy in MS patients is associated with significant side effects, and that Mitoxantrone cumulative dose exposure is clearly associated with cardiac toxicity. Use of Mitoxantrone in worsening RRMS, transitional forms, and in patients no longer responding to first-line therapies should be cautiously considered, and prescribing physicians should: 1) stay on-label, 2) use the lowest possible cumulative dose that achieves the desired clinical effect and 3) vigilantly monitor patients for clinical, cardiac, and neoplastic complications before every dose of Mitoxantrone and annually following treatment.

## Competing interests

VMR declared that there is no conflict of interest. DRJ has received honoraria for speaking and consulting with Bayer, Biogen Idec, Teva, EMD Serono, Pfizer, Novartis, Genzyme, Roche, and Acorda. BWG has participated in speaker’s bureaus and served as a consultant for Biogen Idec, Teva Neuroscience, EMD Serono, Pfizer, Novartis, Genzyme, and Acorda. Excluding Genzyme, BWG has also received grant/research support from the aforementioned companies as well as ITN, Questcor, and Shire. No other industry financial relationships exist. DB was an employee at EMD Serono during the development of this manuscript. DB is currently an employee at BDM Consulting, Inc and declares no other industry financial relationships. FD is an employee of EMD Serono, receiving salary and employment benefits from the company.

## Authors’ contributions

VMR, DRJ, BWG and FD participated in the analysis and interpretation of data. DB contributed to the acquisition of data and performed the statistical analyses. All authors read and approved the final manuscript.

## Pre-publication history

The pre-publication history for this paper can be accessed here:

http://www.biomedcentral.com/1471-2377/13/80/prepub

## Supplementary Material

Additional file 1 Potential cardiac risk explanatory factors: demographic, disease history, dosing (population: all treated subjects). Click here for file

Additional file 2 Serious adverse events reported during the treatment and annual follow-up phases of RENEW. Click here for file

Additional file 3 Serious infection events by relationship to Mitoxantrone during the treatment and follow-up phases of RENEW. Click here for file
